# Epoetin Theta in Anaemic Cancer Patients Receiving Platinum-Based Chemotherapy: A Randomised Controlled Trial

**DOI:** 10.1111/j.1753-5174.2010.00030.x

**Published:** 2010-09

**Authors:** Sergei A Tjulandin, Peter Bias, Reiner Elsässer, Beate Gertz, Erich Kohler, Anton Buchner

**Affiliations:** * Department of Clinical Pharmacology and Chemotherapy, Russian Cancer Research CenterMoscow, Russia; † Department of Clinical Research, Merckle GmbHUlm, Germany; ‡ Department of Biostatistics, Merckle GmbHUlm, Germany; § Department of Preclinical and Clinical ResearchBioGeneriX AG, Mannheim, Germany

**Keywords:** Cancer, Epoetin Beta, Epoetin Theta, Placebo, Symptomatic Anaemia

## Abstract

**Introduction:**

Recombinant human erythropoietin (r-HuEPO) is used to treat symptomatic anaemia due to chemotherapy. A new r-HuEPO, Epoetin theta (Eporatio®), was investigated and compared to placebo and Epoetin beta in a randomised, double-blind clinical trial in adult cancer patients receiving platinum-based chemotherapy, using a fixed weekly starting dose of 20,000 IU Epoetin theta. The primary efficacy endpoint was the responder rate (complete Hb response, Hb increase ≥ 2 g/dL).

**Research Design and Methods:**

223 patients were randomised to s.c. treatment for 12 weeks with either Epoetin theta (*n* = 76) once per week, Epoetin beta (*n* = 73) three times per week or placebo (*n* = 74). The starting dose was 20,000 IU once weekly Epoetin theta or 450 IU/kg_BW_ per week Epoetin beta administered in 3 equal weekly doses.

**Results:**

In the Epoetin theta group were significantly more responders than in the placebo group (65.8 vs. 20.3%, *P* < 0.0001). Epoetin beta was also more effective than placebo (71.2 vs. 20.3%, *P* < 0.0001). The mean weekly dose at the time of complete Hb response was lower in the Epoetin theta group (30,000 IU) than in the Epoetin beta group (42,230 IU). Epoetin theta was clearly more effective than placebo.

**Conclusion:**

This small study showed, that Epoetin theta is a safe and effective treatment of symptomatic anaemia due to platinum-based chemotherapy in cancer patients.

## Introduction

Cancer patients may develop anaemia as a result of disease characteristics, chemotherapy or due to decreased endogenous erythropoietin production. Erythropoietin is the essential growth factor required for the production of red blood cells from late progenitor cells of the erythroid lineage in bone marrow. Patients with reduced or absent endogenous erythropoietin production may need to receive exogenous erythropoietin as replacement therapy for the stimulation of erythropoiesis and correction of their symptomatic anaemia.

Anaemia due to chemotherapy is a major clinical problem and a typical later complication of chemotherapy treatment. The aims of management of this condition are to minimise the use of blood or red cell transfusions, to eliminate symptoms arising from anaemia, to improve quality of life (QoL) and to minimise secondary effects of anaemia. Transfusion is the traditional approach to the management of anaemia. However, growing concerns about infection risks, and the fact that the supply of transfusion products is limited has led to a reduction in the use of red cell transfusions, opening up a need for alternatives to transfusion.

Recombinant human erythropoietin has been used in the treatment of anaemia due to chemotherapy for more than 20 years [1,2]. All epoetins are structurally similar and have the same polypeptide receptor binding sites. However, there are subtle differences in the glycosylation patterns of different types of epoetins.

Epoetin theta which was developed by BioGeneriX AG contains recombinant human erythropoietin (r-HuEPO) as the active drug substance and is structurally similar to other recombinant epoetins.

The objective of this small study was to demonstrate superiority of Epoetin theta compared to placebo for efficacy and to compare the efficacy and safety profile of Epoetin theta with Epoetin beta during the treatment period of 12 weeks in patients with solid tumours receiving platinum-containing chemotherapy.

## Patients and Methods

### Patients

A multinational, multicentre, randomised, placebo- and active-controlled, double-blind phase III study was performed at 54 study centres in 10 countries. The study was conducted according to Good Clinical Practice and the Declaration of Helsinki (1996) and all patients signed informed consent before any study-related activities were performed. The study protocol, amendments, informed consent documents and other relevant study-related documents were reviewed and approved by independent ethics committees of all participating countries (Argentina, Belarus, Brazil, Bulgaria, Croatia, India, Moldova, Romania, Russia, Ukraine). Between October 2005 and July 2007 patients with secondary anaemia related to platinum-containing chemotherapy participated in the study. Male and female patients ≥18 years of age with histologically or cytologically proven diagnosis of a solid tumour were eligible for the study if they gave written informed consent and had anaemia caused by platinum-based chemotherapy defined by a documented Hb concentration of ≤11 g/dL after the last chemotherapy prior to inclusion. Further inclusion criteria among others requested that the patients had at least 1 previous platinum-based chemotherapy cycle as treatment of the current malignancy during the last 4 weeks and had Eastern Cooperative Oncology Group (ECOG) performance status 0, 1, 2 or 3. Exclusion criteria were patients with head and neck tumours, uncontrolled severe hypertension and patients receiving concomitant radiotherapy. Iron substitution was allowed during the study.

### Study Design

**Figure 1:**
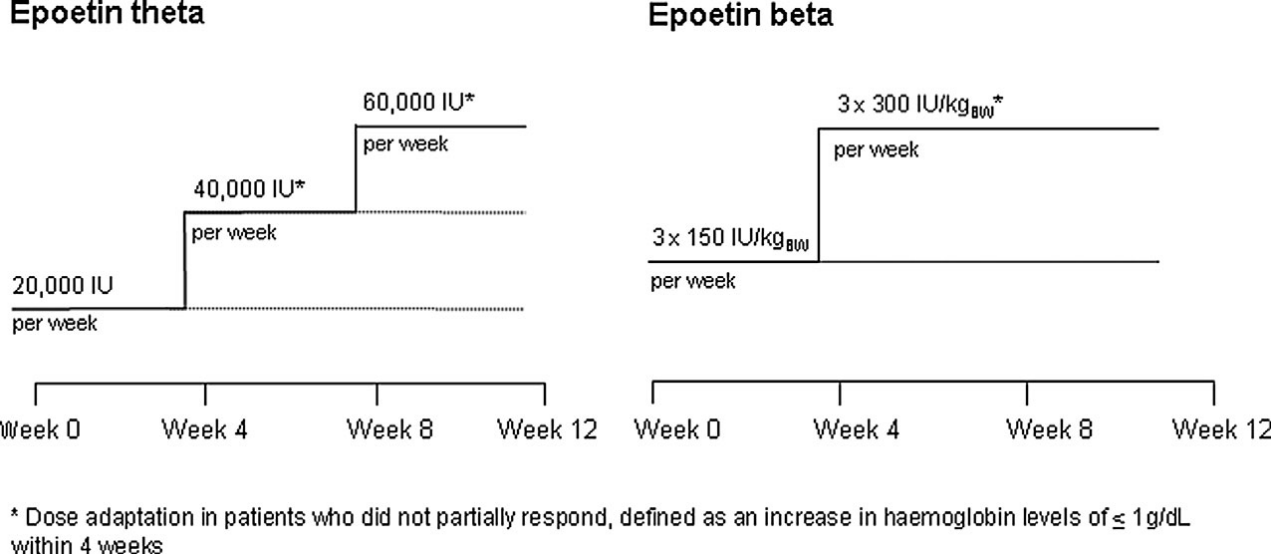
Dose adaptation of Epoetin theta and Epoetin beta.

A total of 223 patients were randomised using a computer-generated allocation schedule in a 1:1:1 ratio stratified by country to double blind treatment for 12 weeks with either Epoetin theta (*n* = 76), Epoetin beta (*n* = 73) or placebo (*n* = 74). The randomisation list was generated by the Department of Biostatistics, Merckle GmbH. Only the person administering study medication was unblinded. All persons assessing outcomes were blinded. Recruitment period was 15 months and the study ended regularly. Patients randomised to Epoetin theta received a starting dose of 20,000 IU Epoetin theta subcutaneously (s.c.) once weekly (e.g., on Mondays) and in addition the same volume of placebo twice weekly (e.g., on Wednesdays and Fridays) for blinding purposes. This starting dose was increased to 40,000 IU/week in patients who did not have a partial response (Hb increase of ≥1 g/dL) after 4 weeks of treatment, and again to 60,000 IU/week in case of insufficient response after the second 4 week period of treatment. If the patient's Hb level increased by more than 2 g/dL in a 4-week period the weekly dose was reduced by 50%. If the Hb level exceeded 13 g/dL the dose was reduced to 50% of the recent dose or was temporarily withhold. Selection of the Epoetin theta doses was based on recent evidence that lower doses than those recommended for authorised products may achieve comparable Hb response, that the same weekly doses administered once or three times weekly result in the same Hb response [3–5] and that fixed doses rather than weight-based doses can be used in anaemic cancer patients [6]. Patients randomised to Epoetin beta (NeoRecormon®, Roche Ltd., United Kingdom) received in accordance with the European product information a starting dose of 450 IU/kg_BW_ per week s.c., administered in 3 equal doses. The dose was doubled to 900 IU/kg_BW_ per week in patients who did not have a partial response after 4 weeks of treatment (Figure 1). As this represents the maximal weekly dose, a second dose increase was not foreseen. Dose reductions took place according to the same schedule as for Epoetin theta. Patients randomised to placebo received s.c. injections of placebo and dose adjustment according to the same schedule as for Epoetin theta for blinding purposes.

Hb, haematocrit and reticulocytes were measured weekly throughout the study. Baseline levels were defined as the mean of two values measured prior to initiation of drug treatment. The FACT-An questionnaire including FACT-G and -F [7] was used to assess the QoL.

Immunogenicity was assessed by a predefined cascade of antibody assays. Confirmed positive samples were investigated for neutralising antibodies in a cellular assay using an erythropoietin dependent UT-7 cell line.

Epoetin theta and Epoetin beta were provided in different containers and the dosing scheme was different between Epoetin theta and Epoetin beta. Therefore, the study drug was prepared and administered by qualified unblinded study personnel who were not involved in any other study assessments. The investigator and all other study personnel were kept blinded and performed all assessments of the patient without knowledge of treatment. An unblinded, independent Data Safety Monitoring Committee closely monitored the safety in order to ensure that patients were not exposed to an unjustifiable risk.

### Endpoints

The pre-specified primary endpoint was the number of patients with a complete Hb response which was defined as an increase in Hb of ≥2 g/dL from baseline without the benefit of a transfusion within the previous four weeks. Secondary efficacy endpoints included the number of patients having a partial Hb response (defined as increase of ≥1 g/dL from baseline), the number of patients having a complete Hb response with the initial dose, the number of patients receiving transfusions, the number of blood units transfused, the time course of Hb, haematocrit and reticulocytes, the QoL (FACT-An, including FACT-G and -F [7]), and the dose of Epoetin theta or Epoetin beta at the time of complete/partial Hb response. Blood transfusions were administered on a case by case basis at the discretion of the investigator. Transfusions should have been avoided at a haemoglobin level ≥8.5 g/dL. Safety endpoints included safety lab, vital signs, incidence of adverse events (AEs) and adverse drug reactions (ADRs), overall tolerability and screening for anti-drug antibodies to Epoetin theta and Epoetin beta at the beginning and end of the study and 60 days after the end of the individual treatment period.

### Statistical Methods

A logistic regression analysis with treatment and baseline haemoglobin value as explanatory variables was performed to estimate the difference in the proportion of complete haemoglobin responders between Epoetin theta and placebo in the confirmatory analysis of the primary efficacy endpoint. The differences between Epoetin beta and placebo and between Epoetin beta and Epoetin theta were estimated with the same statistical model. For the other binary secondary efficacy endpoints the same logistic regression model as for the primary endpoint was estimated, changes of QoL (FACT-scores) from baseline to end of study were compared pairwise among treatment groups with the Wilcoxon-Mann-Whitney test, whereas the treatment groups for the other secondary efficacy endpoints were only compared descriptively. All descriptive tables were displayed by country; no special subgroup analysis was performed.

The sample size calculation required the inclusion of 72 patients per treatment group to achieve a power between 90 and 95% for the statistical superiority test comparing Epoetin theta and placebo (two-sided α = 5%) assuming the actual response rates for Epoetin theta and placebo were 50% and 20% respectively.

## Results

**Figure 2:**
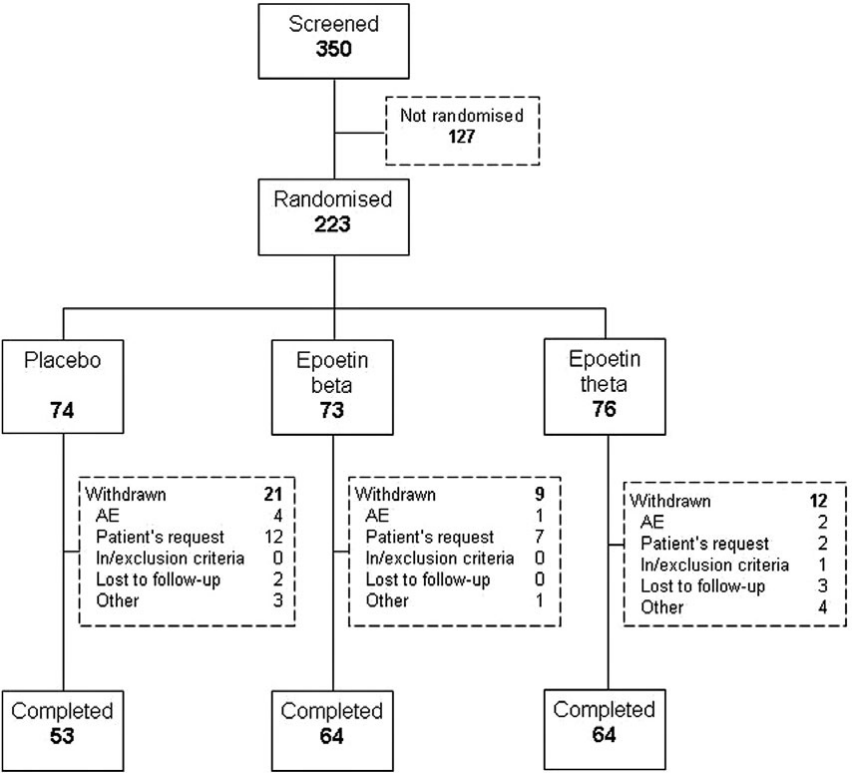
Disposition of patients.

The demographic and baseline characteristics of the 3 treatment groups were comparable. There were no relevant differences between treatment groups with regard to medical history, prior or concomitant medications, ECOG performance status, blood transfusions prior to study entry, concomitant diseases, tumour types and on-study chemotherapies (Table 1). One hundred and eighty-one (81.2%) patients completed the study according to protocol (Figure 2). Of the 42 patients who prematurely discontinued the study, 21 were in the placebo group, 12 were in the Epoetin theta group and 9 were in the Epoetin beta group. The most common reason for discontinuation was patient's request (21 patients), followed by other (unspecified) reasons (8 patients), AEs (7 patients) and, lost to follow-up (5 patients).

**Table 1:** Patient characteristics

	Epoetin theta (*n* = 76)	Epoetin beta (*n* = 73)	Placebo (*n* = 74)	Total (*n* = 223)
Gender [n (%)]				
Male	30 (39.5%)	22 (30.1%)	19 (25.7%)	71 (31.8%)
Female	46 (60.5%)	51 (69.9%)	55 (74.3%)	152 (68.2%)
Age [years]				
Mean ± SD	53.7 ± 10.3	57.3 ± 10.5	57.3 ± 11.5	56.0 ± 10.9
Median	53.5	57.0	59.5	57.0
Range	19.0 to 76.0	28.0 to 83.0	26.0 to 76.0	19.0 to 83.0
Body weight [kg]				
Mean ± SD	66.1 ± 13.2	69.0 ± 14.6	63.0 ± 12.8	66.0 ± 13.7
Median	64.5	66.6	62.1	64.0
Range	35.0 to 97.0	39.0 to 108.7	44.5 to 108.0	35.0 to 108.7
ECOG performance status				
0	6 (7.9%)	9 (12.3%)	5 (6.8%)	20 (9.0%)
1	55 (72.4%)	40 (54.8%)	48 (64.9%)	143 (64.1%)
2	15 (19.7%)	24 (32.9%)	20 (27.0%)	59 (26.5%)
3	0	0	1 (1.4%)	1 (0.4%)
Most common tumour types				
Ovarian epithelial cancer	14 (18.4%)	21 (28.8%)	20 (27.0%)	55 (24.7%)
Gastric cancer	6 (7.9%)	5 (6.8%)	7 (9.5%)	18 (8.1%)
Lung squamous cell carcinoma	4 (5.3%)	5 (6.8%)	7 (9.5%)	16 (7.2%)
Breast cancer	6 (7.9%)	3 (4.1%)	6 (8.1%)	15 (6.7%)
Ovarian epithelial cancer metastatic	6 (7.9%)	6 (8.2%)	3 (4.1%)	15 (6.7%)
Most common on-study CT				
Cisplatin	55 (72.4%)	48 (65.8%)	42 (56.8%)	145 (65.0%)
Carboplatin	22 (28.9%)	29 (39.7%)	24 (32.4%)	75 (33.6%)
Cyclophosphamide	18 (23.7%)	17 (23.3%)	15 (20.3%)	50 (22.4%)
Etoposide	20 (26.3%)	11 (15.1%)	14 (18.9%)	45 (20.2%)

Abbreviations: n = number of patients; SD = standard deviation; CT = chemotherapy.

**Figure 3:**
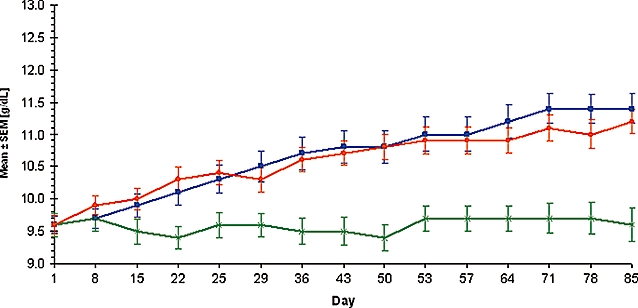
Time course of mean (±SEM) haemoglobin values. Time course of mean (±SEM) haemoglobin values for the full analysis set. Epoetin theta (orange), Epoetin beta (blue) and placebo (green).

The results of efficacy endpoints for the full analysis set are summarised in Table 2 (number of patients with a complete and partial Hb response without blood transfusion, number of patients with a complete Hb response without blood transfusion and with the initial dose, number of patients receiving blood transfusions and number of blood units transfused) and Figure 3 (time course of Hb).

**Table 2:** Results of efficacy endpoints. Number of patients with a complete and partial Hb response without blood transfusion, number of patients with a complete Hb response without blood transfusion and with the initial dose and number of patients receiving blood transfusions for the full analysis set

	Epoetin theta (*n* = 76)	Epoetin beta (*n* = 73)	Placebo (*n* = 74)
Baseline Hb [g/dL] mean ± standard deviation	9.6 ± 1.1	9.5 ± 0.8	9.4 ± 1.2
**Complete Hb response without blood transfusion [n (%)]**	50 (65.8%)	52 (71.2%)	15 (20.3%)
Treatment (Epoetin theta vs. placebo)			
*P* value	<0.0001		
Odds ratio (95% CI)	8.06 (3.89, 17.63)		
Treatment (Epoetin beta vs. placebo)			
*P* value	<0.0001		
Odds ratio (95% CI)	10.25 (4.86, 22.83)		
**Complete Hb response without blood transfusion and dose adjustment [n (%)]**	26 (34.2%)	29 (39.7%)	8 (10.8%)
Treatment (Epoetin theta vs. placebo)			
*P* value	0.0012		
Odds ratio (95% CI)	4.24 (1.84, 10.76)		
Treatment (Epoetin beta vs. placebo)			
*P* value	0.0001		
Odds ratio (95% CI)	5.40 (2.35, 13.68)		
**Partial Hb response without blood transfusion [n (%)]**	69 (90.8%)	66 (90.4%)	37 (50%)
Treatment (Epoetin theta vs. placebo)			
*P* value	<0.0001		
Odds ratio (95% CI)	9.80 (4.19, 26.00)		
Treatment (Epoetin beta vs. placebo)			
*P* value	<0.0001		
Odds ratio (95% CI)	9.39 (4.01, 24.93)		
**Patients received blood transfusions [n (%)]**	8 (10.5%)	9 (12.3%)	18 (24.3%)
Treatment (Epoetin theta vs. placebo)			
*P* value	0.0433		
Odds ratio (95% CI)	0.38 (0.14, 0.95)		
Treatment (Epoetin beta vs. placebo)			
*P* value	0.1042 (n. s.)		

Abbreviations: n = number of patients; Hb = haemoglobin; CI = confidence interval; n. s. = not significant.

### Extent of Exposure

The mean ± SD average weekly dose was higher in the Epoetin beta group compared to the Epoetin theta group (36,973 ± 13,967 vs. 26,425 ± 9,157 IU). This was to be expected as the weekly starting dose of Epoetin beta was 450 IU/kg_BW_. For a patient with a body weight of 69 kg (the mean weight in the Epoetin beta group) the resulting weekly starting dose was 31,050 IU compared to the fixed starting dose of 20,000 IU for the patients treated with Epoetin theta. The mean treatment duration ± SD was comparable in all 3 groups (75.0 ± 16.9 days Epoetin theta vs. 71.0 ± 19.7 days Epoetin beta vs. 70.5 ± 23.7 days placebo).

### Complete Haemoglobin Response without Blood Transfusion

The primary parameter for the confirmatory analysis was the complete Hb response (increase of ≥2 g/dL from baseline) without blood transfusion. The responder rate in the Epoetin theta group was substantially higher than in the placebo group (65.8 vs. 20.3%). The difference between the Epoetin theta and placebo was statistically significant (*P* < 0.0001) with a baseline Hb adjusted odds ratio of 8.06 (95% CI: 3.89, 17.63).

The exploratory analysis for the other group comparisons showed a higher responder rate in the Epoetin beta group vs. the placebo group (71.2 vs. 20.3%, *P* < 0.0001) with a baseline Hb adjusted odds ratio of 10.25 (95% CI: 4.86, 22.83). The difference in treatment effect between Epoetin theta and Epoetin beta was not statistically significant (*P* = 0.5004) with a baseline Hb adjusted odds ratio of 0.79 (95% CI: 0.39, 1.58). Baseline Hb levels had no statistically significant effects on the response rate (*P* = 0.1408).

The proportion of patients who had a complete haemoglobin response without changing the starting dose was significantly higher (*P* = 0.0012; OR: 4.24, 95% CI: 1.84, 10.76) in the Epoetin theta group than in the placebo group (34.2 vs. 10.8%) and was also significantly higher (*P* = 0.0001; OR: 5.40, 95% CI: 2.35, 13.68) in the Epoetin beta group compared to placebo (39.7 vs. 10.8%). The difference between Epoetin theta and Epoetin beta was not statistically significant (*P* = 0.4765; OR: 0.79, 95% CI: 0.40, 1.53).

### Partial Haemoglobin Response without Blood Transfusion

The partial Hb response was defined as increase of ≥1 g/dL from baseline without blood transfusion within the last four week. As for the complete Hb response, the responder rate was higher with Epoetin theta than with placebo (90.8 vs. 50%, *P* < 0.0001, OR: 9.80, 95% CI: 4.19, 26.00) and also higher with Epoetin beta than with placebo (90.4 vs. 50%, *P* < 0.0001, OR: 9.39, 95% CI: 4.01, 24.93). The difference between Epoetin theta and Epoetin beta was not statistically significant (*P* = 0.9394, OR: 1.04, 95% CI: 0.34, 3.20). Baseline Hb levels also had no statistically significant effects on this response rate.

### Number of Patients Receiving Blood Transfusions

More patients in the placebo group than in the Epoetin theta group received blood transfusions (24.3 vs. 10.5%, *P* = 0.0433, OR: 0.38, 95% CI: 0.14, 0.95). The proportion of patients receiving blood transfusions in the Epoetin beta group was also lower than in the placebo group (12.3 vs. 24.3%), however this difference was not statistically significant (*P* = 0.1042, OR: 0.47, 95% CI: 0.18, 1.15). Baseline Hb levels had a statistically significant effect on the rate of blood transfusion (*P* = 0.0005) with an odds ratio of 0.53 (95% CI: 0.37, 0.75) per g/dL baseline Hb comparing Epoetin theta with placebo.

### Number of Blood Units Transfused

The mean ± SD number of blood units transfused after randomisation in those patients who needed a transfusion was 1.8 ± 0.7 units in the Epoetin beta group, 2.8 ± 2.9 units in the placebo group and 3.3 ± 2.2 units in the Epoetin theta group.

### Time Course of Haemoglobin, Haematocrit and Reticulocytes

Baseline Hb values were similar in all 3 treatment groups (Figure 3). Over the course of the study mean Hb levels rose steadily in the Epoetin theta and Epoetin beta groups, reaching 10 g/dL by day 15 in the Epoetin theta group and by day 22 in the Epoetin beta group. In the Epoetin theta group, mean ± SD Hb levels rose from 9.6 ± 1.1 g/dL at baseline to 11.2 ± 1.6 g/dL at the end of the study, an increase of 1.6 g/dL. In the Epoetin beta group, mean ± SD Hb levels rose from 9.5 ± 0.8 g/dL at baseline to 11.4 ± 2.0 g/dL at the end of the study, an increase of 1.9 g/dL. In the placebo group, there was no consistent change in Hb levels over the course of the study and the mean level never reached 10 g/dL. Mean ± SD Hb levels were 9.4 ± 1.2 g/dL at baseline and 9.6 ± 2.0 g/dL at the end of the study.

The changes of haematocrit values were very similar to the changes of Hb values over time. Absolute reticulocyte values showed a high degree of variability in all 3 treatment groups and at all timepoints.

### Dose of Epoetin Theta or Epoetin Beta at the Time of Partial or Complete Haemoglobin Response

The mean ± SD weekly dose of Epoetin theta at the time of complete Hb response was 30,000 ± 12,936 IU, and at the time of partial Hb response it was 27,826 ± 12,469 IU. A dose of up to 20,000 IU was sufficient for complete Hb response in 52% of patients with complete response in the Epoetin theta dose group. A dose of between 20,000 and 40,000 IU of Epoetin theta was sufficient to achieve complete Hb response in a further 40% of patients with complete response. The mean ± SD weekly dose of Epoetin beta at the time of complete Hb response was 42,230 ± 23,455 IU, and at the time of partial Hb response it was 39,827 ± 21,831 IU. In the Epoetin beta group 55.8% of patients with complete response achieved this with the starting dose.

### Safety Evaluation

The overall frequencies of adverse events (AEs) were 76.3% in the Epoetin theta group, 85.1% in the placebo group, and 86.3% in the Epoetin beta group, see Table 3. The high incidence of AEs was to be expected in cancer patients receiving chemotherapy. The overall profile of the most frequently reported AEs can be expected in the rather elderly study population of cancer patients receiving platinum-based chemotherapy. Frequencies only exceeded 10% for nausea (33.2%), neutropoenia (22.9%), asthenia (22.4%), vomiting (18.4%), thrombocytopenia (16.6%) and leukopenia (16.1%). The incidence of skin reactions that might have been caused by the subcutaneous administration of study medication was low and comparable in all groups: 3 in Epoetin beta patients and 1, each, in placebo and Epoetin theta patients. The incidence of hypertension was 2.6% in the Epoetin theta group and 2.7% in each of the other two treatment groups.

**Table 3:** Frequencies of TEAE categories (full analysis set)

	Epoetin theta (*n* = 76)		Epoetin beta (*n* = 73)		Placebo (*n* = 74)	
			
Category of TEAE	n	%	n	%	n	%
Any TEAE	58	76.3	63	86.3	63	85.1
Related TEAE = TEADR	14	18.4	16	21.9	13	17.6
Serious TEAE	9	11.8	9	12.3	15	20.3
Serious TEADR	1	1.3	1	1.4	0	-
Death	5	6.6	4	5.5	12	16.2

Abbreviations: n = number of patients; multiple mentions per patient are possible.

Adverse drug reactions (ADRs) with a causal relationship to the study medication as assessed by the investigator were reported in 14 (18.4%) patients in the Epoetin theta group, 13 (17.6%) patients in the placebo group, and 16 (21.9%) patients in the Epoetin beta group. The most common ADRs were nausea (8.5%), asthenia (5.8%), vomiting (4.5%) and headache (3.6%). All of these events commonly occur in cancer patients receiving platinum-based chemotherapy.

Twenty-one of the patients in this study (5 Epoetin theta, 12 placebo, 4 Epoetin beta) died during the study period. The most frequent reason for death was disease progression (1 Epoetin beta, 6 placebo, 3 Epoetin theta). Serious adverse events (SAEs) were reported in 33 (14.8%) patients (9 Epoetin theta, 15 placebo, 9 Epoetin beta). AEs leading to discontinuation of study were reported in 13 patients (4 Epoetin theta, 6 placebo, 3 Epoetin beta).

Results for safety lab variables, vital signs, body weight, 12-lead ECG, physical examination, tolerability, skin irritation, and results of current chemotherapy did not give rise to any safety concerns.

Tolerability as assessed by the patients was very good or good in 89.3%, 76.4%, and 90.3% of patients in the Epoetin theta, placebo, and Epoetin beta group. Tolerability as assessed by the investigators was very good or good in 93.3%, 88.9%, and 93.1% of patients, respectively.

Response rates (complete plus partial response) to chemotherapy were comparable in the Epoetin theta and placebo groups (31.6% vs. 29.7%), therefore there is no evidence that Epoetin theta could have had a negative impact on the course of the disease.

The incidence of anti-drug antibodies to Epoetin theta and Epoetin beta was assessed at the beginning and end of the study and 60 days after the end of the individual treatment period. None of the patients enclosed in the study developed neutralising anti-epoetin antibodies to Epoetin theta or Epoetin beta.

## Discussion

The selection of the weekly fixed starting dose of 20,000 IU Epoetin theta, which is lower than the recommended starting dose of other epoetins, is in line with recent recommendations for the treatment of cancer patients with epoetins, i.e., to use the lowest dose needed to avoid red blood cell transfusion [8].

A meta-analysis based on 14 randomised controlled trials including 2,347 cancer patients, with haematologic response to treatment with erythropoietin defined as an increase in Hb of at least 2 g/dL unrelated to transfusion, reported response rates of 9–70% [9]. The corresponding relative risk (RR) for haematologic response in the erythropoietin group was 3.60 (95% CI: 3.07, 4.23). Thus, patients receiving an erythropoietin are 3 to 4 times more likely to achieve Hb response. The response rates of 65.8% in the Epoetin theta group and 20.3% in the placebo group observed in this study therefore compare favourably with the literature, although the weekly starting dose was fixed and not body weight adjusted and lower than the weekly dose in the studies included in the above meta-analysis.

A meta-analysis based on 25 randomised controlled trials including 3,069 cancer patients showed that the use of erythropoietin significantly reduced the relative risk of red blood cell transfusion (RR 0.67, 95% CI: 0.62, 0.73) [9]. The results of the present study compare favourably with information derived from the literature. This holds true also for the further efficacy parameters like the haemoglobin and haematocrit levels over time. Of note, the mean weekly dose at the time of complete Hb response was substantially lower in the Epoetin theta group (30,000 IU) than in the Epoetin beta group (42,230 IU) and 52% of the complete responders in the Epoetin beta group achieved the response with the starting dose of 20,000 IU. This result confirms that the Epoetin theta dosing schedule tested here is suitable for an effective and well tolerated treatment of anaemic cancer patients.

The frequencies and type of AEs as observed in this study were to be expected in cancer patients receiving chemotherapy. The results indicate that Epoetin theta generally showed a good tolerability with an AE or ADR incidence not higher than in the placebo group.

The number of fatalities was highest in the placebo group in this study. Furthermore no anti-drug antibodies to Epoetin theta and Epoetin beta were detected in any patient of this study.

Results for other safety parameters like SAEs, AEs leading to discontinuation, safety lab variables, vital signs, etc. did not give rise to any safety concerns.

It should be taken into consideration that the relatively small number of patients per treatment group was not enough to detect rare adverse events. For a detailed assessment of safety, further patients would be needed.

## Conclusions

In this small study, Epoetin theta with a weekly starting dose of 20,000 IU is superior to placebo in terms of complete Hb response without blood transfusion. Epoetin theta is a safe and effective treatment for the treatment of anaemia due to platinum-based chemotherapy in patients with solid tumours.
